# Positive Feedback Loop of SNAIL-IL-6 Mediates Myofibroblastic Differentiation Activity in Precancerous Oral Submucous Fibrosis

**DOI:** 10.3390/cancers12061611

**Published:** 2020-06-18

**Authors:** Chih-Yu Peng, Yi-Wen Liao, Ming-Yi Lu, Chieh-Mei Yang, Pei-Ling Hsieh, Cheng-Chia Yu

**Affiliations:** 1 School of Dentistry, Chung Shan Medical University, Taichung 40201, Taiwan; cyp@csmu.edu.tw (C.-Y.P.); rabbity0225@gmail.com (Y.-W.L.); miexyz@gmail.com (M.-Y.L.); mayfire0427@hotmail.com (C.-M.Y.); 2 Department of Dentistry, Chung Shan Medical University Hospital, Taichung 40201, Taiwan; 3 Department of Anatomy, School of Medicine, China Medical University, Taichung 40402, Taiwan; 4 Institute of Oral Sciences, Chung Shan Medical University, Taichung 40201, Taiwan

**Keywords:** oral submucosal fibrosis, arecoline, Snail, interleukin-6, myofibroblast

## Abstract

Oral submucosal fibrosis (OSF) is a premalignant disorder of the oral cavity, and areca nut chewing is known to be a major etiological factor that could induce epithelial to mesenchymal transition (EMT) and activate buccal mucosal fibroblasts (BMFs). However, this detailed mechanism is not fully understood. In this study, we showed that the upregulation of Snail in OSF samples and fibrotic BMFs (fBMFs) may result from constant irritation by arecoline, a major alkaloid of the areca nut. The elevation of Snail triggered myofibroblast transdifferentiation and was crucial to the persistent activation of fBMFs. Meanwhile, Snail increased the expression of numerous fibrosis factors (e.g., α-SMA and collagen I) as well as IL-6. Results from bioinformatics software and a luciferase-based reporter assay revealed that IL-6 was a direct target of Snail. Moreover, IL-6 in BMFs was found to further increase the expression of Snail and mediate Snail-induced myofibroblast activation. These findings suggested that there was a positive loop between Snail and IL-6 to regulate the areca nut-associated myofibroblast transdifferentiation, which implied that the blockage of Snail may serve as a favorable therapeutic strategy for OSF treatment.

## 1. Introduction

Oral submucosal fibrosis (OSF) is an insidious inflammatory condition which accompanies the excess fibrous connective tissue which accumulates in the oral cavity. Patients often suffer from restricted mouth opening, which severely compromises their quality of life due to its adverse effects on chewing, oral hygiene and social activities. Most importantly, insufficient exposure of the intraoral cavity for cancer surveillance may potentially delay appropriate management. It has been suggested that OSF is one of the most potentially malignant oral disorders [1] and around 10% of OSF cases may transform into malignancy, particularly oral squamous cell carcinoma (OSCC) [2,3]. An epidemiological study has revealed that the habit of areca nut chewing is a major etiology [4], and areca nut constituents have been known to increase pro-inflammatory cytokines, such as transforming growth factor (TGF)-β [5]. It has been indicated that TGF-β stimulates the activation of pro-collagen genes and the elevation of collagenase inhibitors, which disturbs the collagen metabolism in OSF [6]. Besides this, TGF-β has been shown to regulate the differentiation of oral fibroblasts into myofibroblasts [7].

The activated myofibroblasts have been implicated in the excessive deposition of extracellular matrix (ECM) components [8]. The expression of alpha-smooth muscle actin (α-SMA) is one of the distinct features of the fully differentiated myofibroblasts [9], and an increased α-SMA expression is sufficient to enhance fibroblast contractile activity [10]. Apart from TGF-β, it has been shown that other pro-inflammatory cytokines also play critical roles in the persistent activation of myofibroblasts. Earlier studies have demonstrated the pro-fibrotic role of interleukin (IL)-6 in α-SMA induction [11] and the transition of fibroblasts into myofibroblast phenotypes [12]. Recently, it has been revealed that IL-6 could augment TGF-β/Smad signaling [13] and participate in the TGF-β1-mediated transdifferentiation of myofibroblasts [14]. Furthermore, accumulating evidence has indicated that IL-6 is a potent inducer of epithelial to mesenchymal transition (EMT) in various carcinomas via the signal transducer and activator of transcription 3 (Stat3)/Snail signaling pathway [15,16]. It is well known that EMT is a potential source of myofibroblasts as well as a crucial process for carcinogenesis [17]. Numerous EMT transcriptional factors, such as ZEB-1 and Twist, were found to be involved in the transdifferentiation of myofibroblast in areca nut-associated OSF [18,19]. 

Snail is the first discovered transcriptional repressor of E-cadherin in the Snail superfamily, and the hallmark of EMT is the downregulation of E-cadherin [20]. Members of this family share similar structures, with a unique conserve motif near the zinc fingers in the C-terminal domain, which mediates sequence-specific interactions with promoters containing an E-box sequence on the target genes [20]. It has been shown that Snail is sufficient to repress epithelial genes like E-cadherin by binding to E-box sequences through the zinc-finger domains [21]. As a matter of fact, the elevated expression of Snail has been found in various types of fibrosis [22,23] and our previous study revealed that Snail was able to bind to the E-box in the α-SMA promoter in buccal mucosal fibroblasts (BMFs) [24]. Moreover, it has been shown that arecoline increased Snail expression in human oral keratinocytes and the overexpression of Snail was proven to be associated with the clinico-pathologic features of OSCCs [25]. However, the relationship between pro-inflammatory cytokines and the Snail-mediated transdifferentiation of myofibroblasts in OSF remains unclear.

In an effort to elucidate the role of Snail in the activation of myofibroblasts, we first examined the expression of Snail in OSF tissues and fibrotic buccal mucosal fibroblasts (fBMFs) derived from OSF specimens. We specifically silenced the expression of Snail in arecoline-treated normal buccal mucosal fibroblasts (BMFs), followed by an examination of myofibroblast activities, and we confirmed the findings with fibrotic BMFs (fBMFs). Next, we investigated whether Snail affected the expression of fibrosis factors and our results showed that IL-6 was a direct target of Snail. Furthermore, we found that the inhibition of IL-6 not only downregulated a number of fibrosis markers (e.g., α-SMA and type I collagen) but also suppressed the expression of Snail, indicating that IL-6 and Snail may enhance or amplify each other in the modulation of myofibroblast activation. Altogether, these findings provided insights into the involvement of the positive feedback loop of Snail-IL-6 in the pathogenesis of OSF.

## 2. Results

### 2.1. Elevation of Snail Is Observed in the Areca Nut Chewing-Associated OSF

**Figure 1:**
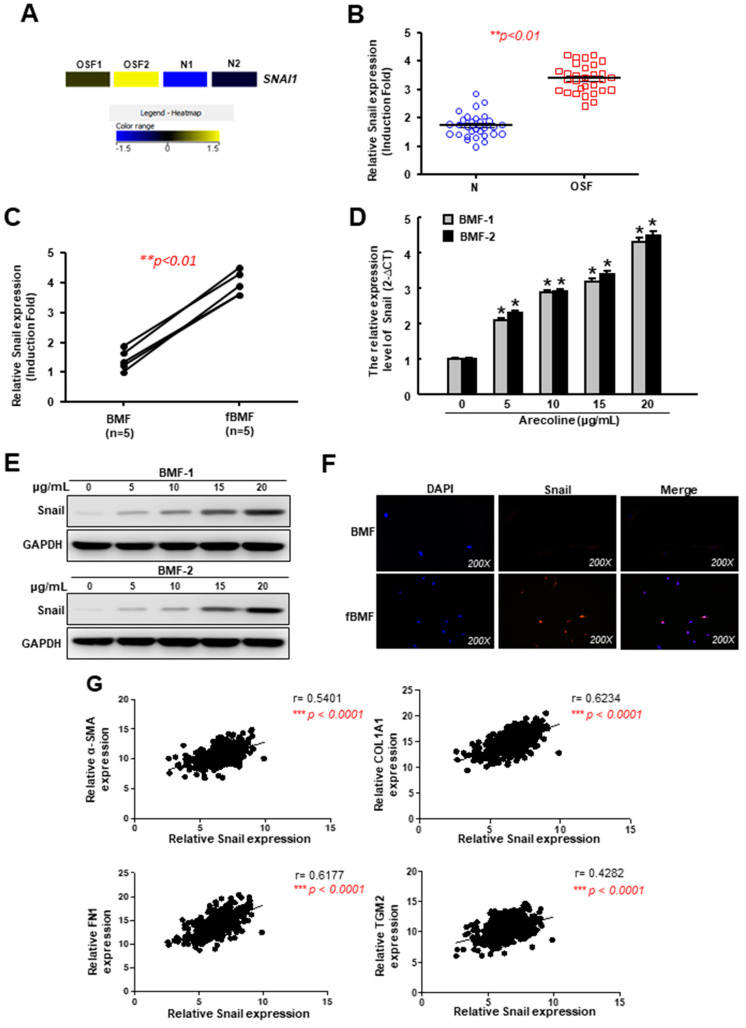
The expression of Snail is upregulated in oral submucosal fibrosis (OSF) tissues owing to arecoline stimulation. (**A**) A heat-map showing the upregulation of Snail in OSF (*n =* 2) and normal (*n =* 2) buccal mucosal tissues using RNA-sequencing; (**B**) The relative expression of Snail in normal and OSF specimens (*n =* 30); (**C**) The relative expression of Snail in normal human buccal mucosal fibroblasts (BMFs) and fibrotic BMFs (fBMFs) derived from OSF tissues; (**D**) Gene and (**E**) protein expression levels of Snail in BMFs treated with various concentration of arecoline; (**F**) Immunofluorescent staining of Snail in BMFs and fBMFs; (**G**) Analysis of the relationship between Snail and various fibrosis factor, including α-SMA, type I collagen alpha-1, fibronectin and tissue transglutaminase using oral squamous cell carcinoma (OSCC) data from The Cancer Genome Atlas (TCGA). Results are means ± SD of triplicate samples from three experiments.

We used RNA-sequencing to identify differentially expressed genes between two OSF tissues and two normal buccal mucosa. We found that one of the most abberantly expressed genes was Snail, which was upregulated in OSF tissues compared to the normal buccal mucosa (Figure 1A). Results from the RT-PCR confirmed that the relative expression of Snail in OSF tissues (Figure 1B) and fBMFs derived from OSF specimens (Figure 1C) were indeed increased. Next, we treated human BMFs with various concentrations of arecoline (an areca nut alkaloid) and assessed the alteration of Snail. Arecoline has been proven to stimulate BMF activation and ECM component accumulation [26]. Our results showed that the gene (Figure 1D) and protein (Figure 1E and Figure S1) expression levels of Snail in BMFs were dose-dependently elevated in response to the arecoline treatment. The immunofluorescent image revealed that Snail translocated into the cell nuclei of the fBMFs in order to exert its function of gene regulation [27], while the localization of Snail remained in the cytoplasm of normal BMFs (Figure 1F). Besides this, we analyzed the data of OSCC tissues from The Cancer Genome Atlas (TCGA) and found that there was a positive relationship between Snail and the myofibroblast marker α-SMA (Figure 1G). In addition, Snail was also positively correlated with type I collagen and fibronectin (Figure 1G), which have been shown to be less phagocytosed in arecoline-treated BMFs [28]. Another factor that was regulated by arecoline in oral fibroblasts, transglutaminase-2 [29], was associated with Snail as well (Figure 1G). These findings suggest that arecoline-increased Snail may contribute to the progression of OSF and even its transformation into malignancy. 

### 2.2. Snail Is Critical to Arecoline-Induced Myofibroblast Transdifferentiation

**Figure 2:**
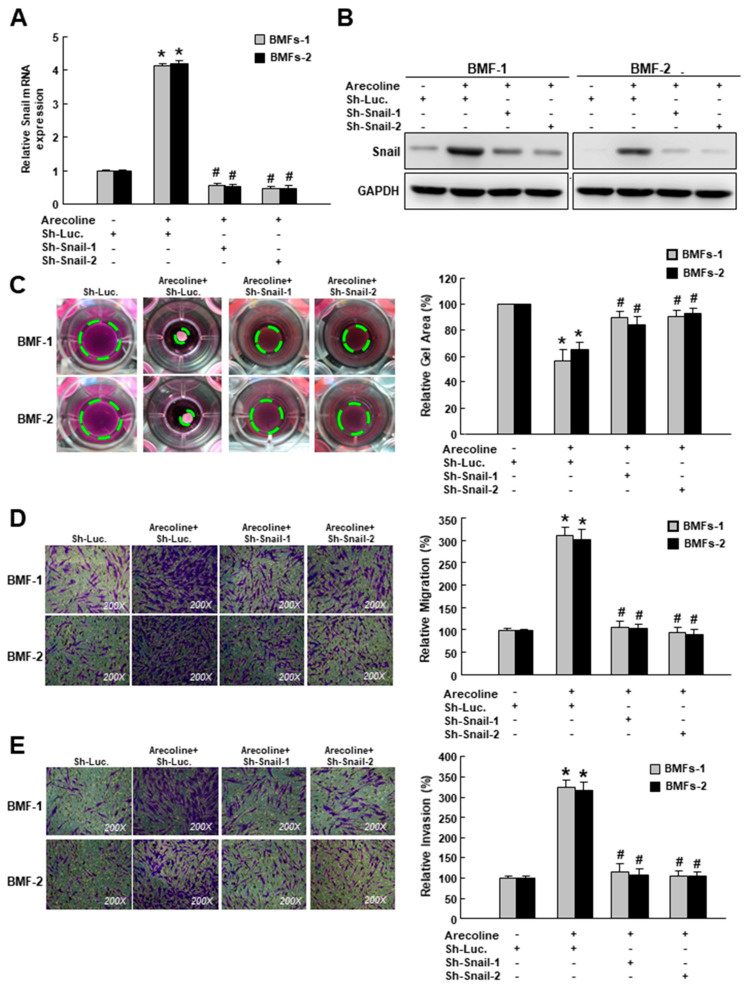
Snail is essential for arecoline-induced myofibroblast transdifferentiation. (**A**) Relative gene and (**B**) protein expression levels of Snail in arecoline-treated BMFs with sh-Luc or sh-Snail; myofibroblast activities, including (**C**) collagen gel contraction, (**D**) migration and (**E**) invasion abilities of arecoline-treated BMFs with sh-Luc or sh-Snail. The area of the gel is indicated by the green dotted line. Results are means ± SD of triplicate samples from three experiments. * *p* < 0.05 compared to sh-Luc group. ^#^ *p* < 0.05 compared to arecoline+ sh-Luc group.

To investigate whether Snail participated in the arecoline-stimulated myofibroblast activation, a small hairpin RNA was employed to suppress the expression of Snail. After confirming the arecoline-induced gene (Figure 2A) and protein (Figure 2B) expression of Snail were inhibited in BMFs transduced with sh-Snail, we examined the following myofibroblast activities. We demonstrated that the area of the arecoline-increased collagen gel contraction (green dotted line) was diminished in cells with sh-Snail (Figure 2C). Additionally, the enhanced migration and invasion capabilities in the arecoline-stimulated BMFs were prevented by the knockdown of Snail (Figure 2D,E and Figure S2). These results demonstrated that Snail was essential to arecoline-induced myofibroblast transdifferentiation.

**Figure 3:**
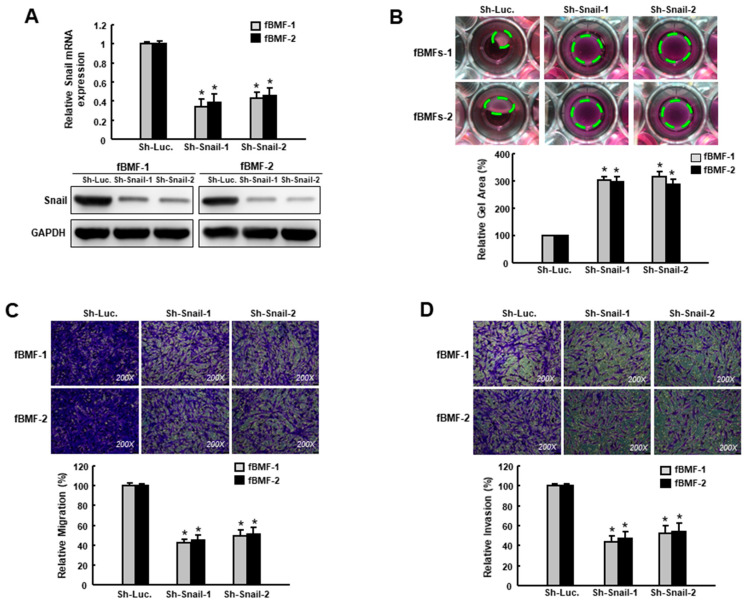
Suppression of Snail inhibits the characteristics of myofibroblasts. (**A**) Gene and protein expression of Snail in fBMFs transduced with sh-Luc or sh-Snail; (**B**) Collagen gel contractility, (**C**) migration and (**D**) invasion of fBMFs transduced with sh-Luc or sh-Snail. The area of the gel is indicated by the green dotted line. Results are means ± SD of triplicate samples from three experiments. * *p* < 0.05 compared to sh-Luc group.

In order to examine the effect of Snail inhibition on the suppression of myofibroblast behaviors, we conducted a loss-of-function experiment on the fBMFs (Figure 3A, Figures S3 and S4). The collagen contractility (Figure 3B), migration (Figure 3C) and invasion (Figure 3D) abilities were all downregulated, indicating that Snail was crucial in the maintenance of myofibroblast phenotypes, and the repression of Snail was able to reduce the myofibroblast characteristics of fBMFs.

**Figure 4:**
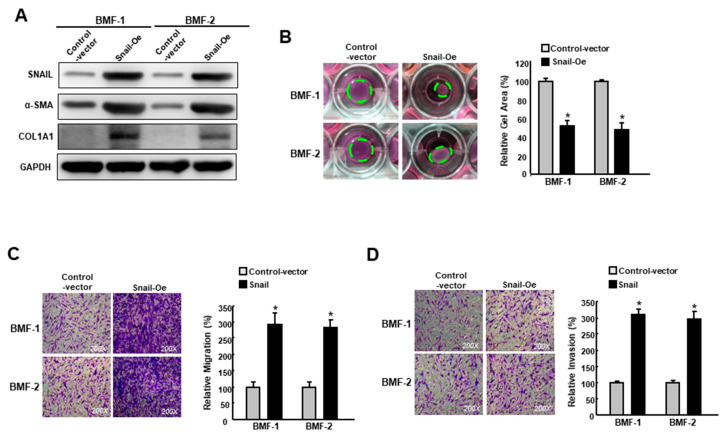
Overexpression of Snail enhances the features of myofibroblasts. (**A**) The protein expression level of Snail, α-SMA and type I collagen in BMFs overexpressing Snail or control vector; (**B**) Collagen gel contraction, (**C**) migration and (**D**) invasion capacities in BMFs overexpressing Snail or control vector. The area of the gel is indicated by the green dotted line. Results are means ± SD of triplicate samples from three experiments. * *p* < 0.05 compared to control vector.

To further verify its fibrotic role in BMFs, the ectopic expression of Snail was carried out in two BMFs (Figure 4A, Figures S5 and S6). A Western blot analysis showed that fibrosis markers, including α-SMA and type I collagen, were elevated in Snail-overexpressing BMFs (Figure 4A). As expected, myofibroblast activities, including collagen contractility (Figure 4B), migration (Figure 4C) and invasion (Figure 4D) capacities, were increased. Taken together, these findings depicted the pivotal role of Snail in the induction and maintenance of myofibroblast transdifferentiation.

### 2.3. The Interplay Between IL-6 and Snail Contributes to the Transdifferentiation of Myofibroblasts

**Figure 5:**
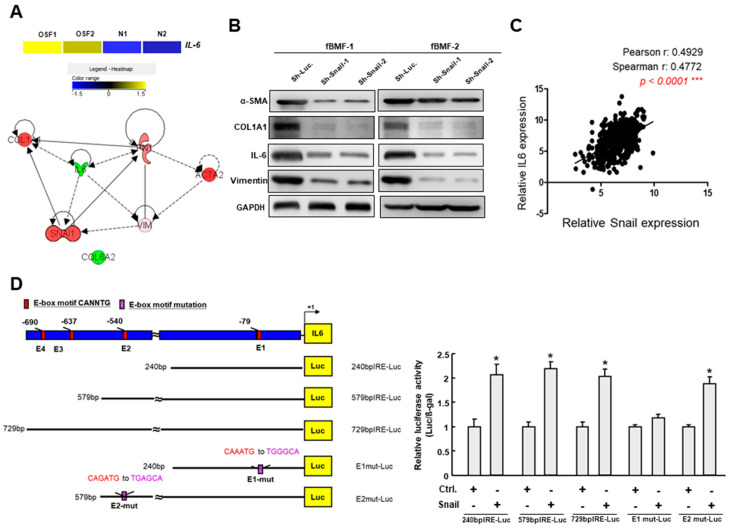
Snail directly binds to the IL-6 promoter via its E-box. (**A**) RNA sequencing analysis showed that IL-6 was upregulated in OSF tissues compared to the normal buccal mucosa (upper panel); Predicted interacting factors of Snail using bioinformatics software (lower panel); (**B**) Protein expression of several fibrosis factors, including α-SMA, type I collagen, IL-6 and vimentin, in fBMFs transduced with sh-Luc or sh-Snail; (**C**) Analysis of the relationship between Snail and IL-6 using OSCC data from The Cancer Genome Atlas (TCGA); (**D**) Schematic representation of E-box domain in IL-6 promoter region and reporter constructs. The full-length, deletion and mutated promoter reporter constructs were designed and subjected to a luciferase-based reporter assay.

Subsequently, we utilized bioinformatics software to predict the interacting factors of Snail and identified several potential targets (Figure 5A). IL-6 was also upregulated in the OSF tissues compared to the normal buccal mucosa by RNA-seq analysis (Figure 5A). The results of the Western blot revealed that silencing Snail reduced the expression of α-SMA, type I collagen, IL-6 and vimentin in fBMFs (Figure 5B and Figures S7–S11), which was in accordance with the results shown in Figure 1G and our recent findings that Snail was able to bind to the E-box in the α-SMA promoter [24]. Among these targets, we noticed that another factor, IL-6, may also be regulated by Snail via binding to the E-box region in the promoter of IL-6. After analyzing the OSCC data from TCGA, we found that there was a positive correlation between Snail and IL-6 (Figure 5C). Hence, we performed a luciferase-based reporter assay to elucidate whether the IL-6 promoter can be activated by Snail. The reporter constructs containing wild-type, mutated and deleted lengths of the IL-6 promoter are shown in Figure 5D. An increase in IL-6 promoter activity was observed after transient transfection with Snail (Figure 5D, the “240 bp” group, “579 bp” group and “729 bp” group), while deleting a region containing the E1 box hampered IL-6 activation.

**Figure 6:**
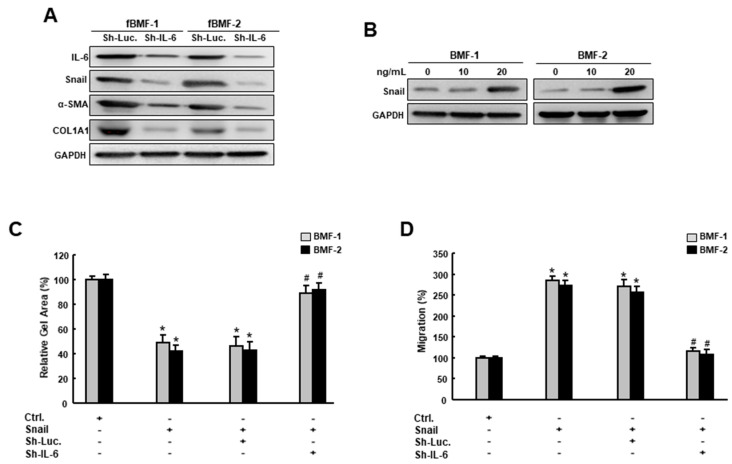
IL-6 is essential to the Snail-induced myofibroblasts’ features. (**A**) Protein expression of IL-6, Snail, α-SMA and type I collagen in fBMFs transduced with sh-Luc or sh-IL-6; (**B**) Protein expression of Snail in BMFs treated with various concentrations of IL-6; (**C**) Collagen gel contractility and (**D**) migration capacity in BMFs with an overexpression of Snail with sh-Luc or sh-IL-6. Results are means ± SD of triplicate samples from three experiments. * *p* < 0.05 compared to ctrl. group. ^#^ *p* < 0.05 compared to Sh-Luc +Snail group.

**Figure 7:**
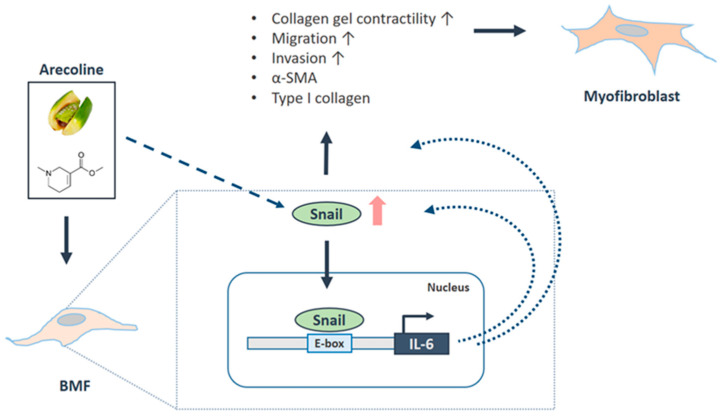
Schematic diagram of the role of Snail in OSF progression. Arecoline, a major alkaloid from the areca nut, activates the transdifferentiation of buccal mucosal fibroblasts (BMF) into myofibroblasts (including the increased phenotypes and fibrosis markers) via the upregulation of Snail. Besides this, the arecoline-induced Snail directly binds to the promoter of IL-6, which further augments the expression of Snail and mediates the Snail-induced myofibroblast activation. Altogether, these findings demonstrate that arecoline triggered a positive feedback loop for Snail and IL-6 to promote the oral fibrogenesis.

On the other hand, it has been shown that IL-6 promotes head and neck tumor metastasis by inducing EMT [16]. Consequently, we examined the effect of IL-6 inhibition on the expression of Snail and other fibrosis markers. Our results showed that Snail, α-SMA and type I collagen were all downregulated in the fBMFs that received sh-IL-6 (Figure 6A, Figures S12 and S13). Moreover, the delivery of recombinant IL-6 in BMFs increased the expression of Snail in a dose-dependent fashion (Figure 6B and Figure S14), suggesting that the expression of IL-6 affected the upregulation and downregulation of Snail. In Figure 6C,D, we have demonstrated that the overexpression of Snail increased the myofibroblast activities (collagen gel contractility and migration ability), whereas the knockdown of IL-6 reversed these Snail-induced phenomena. Collectively, we demonstrated that IL-6 and Snail regulate the expression of each other in order to modulate myofibroblast transdifferentiation (Figure 7).

## 3. Discussion

It is widely accepted that chronic inflammation is associated with pathological fibrosis. Although it has been demonstrated that arecoline contributes to the increased secretion of inflammatory cytokines and the persistent activation of myofibroblasts [5], the underlying mechanisms of the arecoline-induced inflammation have not been fully unraveled. In the present study, we showed that the expression of Snail was elevated in OSF tissues, which may due to the constant irritation caused by a major areca nut constituent (arecoline). Moreover, our results demonstrated that Snail was able to directly bind to IL-6 and the downregulation of Snail abrogated the expression of IL-6 in fBMFs (Figure 5). Given that the elevated expression of IL-6 in OSF tissues has been reported previously [30], our observation of the upregulated Snail caused by arecoline and Snail-regulated IL-6 expression provided a possible mechanism underlying the areca nut-increased inflammation in the oral mucosa.

In addition to TGF-β, IL-6 has also been shown to modulate α-SMA expression in dermal fibroblasts [11]. Besides this, incubation with IL-6 and sIL-6R have been shown to increase type I collagen in healthy dermal fibroblasts in vitro [31]. In this study, we showed that the inhibition of IL-6 was able to reduce the expression of α-SMA and type I collagen in fBMFs. Moreover, we demonstrated that the knockdown of IL-6 lessened the expression of Snail and the administration of IL-6 amplified Snail expression. In the last experiment (Figure 6C,D), we demonstrated that the knockdown of IL-6 hindered the myofibroblast activities in Snail-overexpressing cells. In addition to the direct binding of Snail to α-SMA [24], these results indicated that sufficient IL-6 was also required for Snail to induce myofibroblast activities. Along with the findings of Snail-regulated myofibroblast phenotypes, we showed that IL-6 may exert its fibrosis ability and elicit myofibroblast transdifferentiation via the modulation of numerous fibrosis factors and Snail in oral fibroblasts.

Mounting evidence has shown that epithelial cells can give rise to fibroblasts and thereby contribute to fibrosis via EMT [32]. Our previous work has demonstrated that several EMT transcriptional factors, such as ZEB-1 and Twist, are associated with the transdifferentiation of myofibroblast in OSF [18,19]. As a potent EMT inducer, Snail has been suggested to be involved in the pathological fibrogenesis. An increased level of Snail has been observed in various types of tissue fibrosis [22,23], including OSF (Figure 1). An overexpression of Snail was shown to increase the expression of fibronectin and the connective tissue growth factor (CTGF) [22,33], while the suppression of Snail reduced fibrosis [34]. These findings were in agreement with our results that Snail was positively associated with various fibrosis factors (e.g., type I collagen and fibronectin) and the reduced myofibroblast activities in fBMFs with sh-Snail. Aside from stimulating myofibroblast activation via the Snail–CTGF axis [23], Snail also has been found to promote collagen production through increased TGF-β signaling [35]. In OSF, as our recent work has shown, Snail is able to bind to the promoter of α-SMA [24], and the present study demonstrated that Snail can increase the production of IL-6. The overall findings, ours and those of others, imply that Snail may stimulate myofibroblast activation through the direct binding of α-SMA and the enhancement of pro-fibrotic signaling (e.g., CTGF, IL-6 and TGF-β).

The expression and function of Snail were regulated by various biochemical and mechanical extracellular stimuli. For instance, it has been suggested that Snail is activated by the TGF-β/ Smad3 pathway [36] and is required for TGF-β-induced EMT [37]. TGF-β has been postulated as one of the main causative events for the induction of myofibroblast transdifferentiation in OSF [5] and may be a possible mechanism of the upregulated Snail. Glycogen synthase kinase-3beta (GSK-3β) was found to be another upstream modulator and an endogenous inhibitor of Snail [38,39]. It has been considered as a tumor suppressor in oral cancer [40] and its reduced expression is related to lower survival in OSCC [41]. Nevertheless, it has been reported that areca nut extracts can upregulate the phosphorylation of GSK-3β in neutrophils [42] and OSCCs [43]. Hence, whether GSK-3β participates in the regulation of Snail in areca nut-associated OSF remains to be determined. The other probable contributor to the elevated Snail in OSF is IL-6 since IL-6 has been shown to induce Snail expression in tumor cells and promote metastasis [15,16], and our results showed that the expression of Snail was indeed affected by IL-6. Several studies have revealed the IL-6-driven EMT process in various types of cancers. It has been demonstrated that IL-6 induces the expression of Snail through JAK/Stat3 signaling in cervical [15], colorectal [44] or head and neck cancers [16]. It was likely that IL-6 mediated the Snail expression in the present study through Stat3, although we have not verified the significance of Stat3 here. Overall, we demonstrated that the downregulation of IL-6 diminished the expression of Snail in fBMFs, suggesting that the repression of IL-6 may alleviate the progression of OSF. On the other hand, the administration of IL-6 dose-dependently induced the expression of Snail in BMFs, indicating that areca nut-increased IL-6 may provoke the activation of myofibroblasts, leading to oral fibrogenesis.

## 4. Materials and Methods

### 4.1. Chemicals

Arecoline (an alkaloid from areca nut) and collagen solution from bovine skin were purchased from Sigma-Aldrich (St. Louis, MO, USA). Arecoline was employed in order to induce myofibroblast transdifferentiation and collagen was used in the collagen gel contraction assay.

### 4.2. Tissue Acquisition and Cell Culture

In this study, all procedures performed which involved human participants were in accordance with the tenets of the Declaration of Helsinki and reviewed by the Institutional Review Committee at Chung Shan Medical University, Taichung, Taiwan. A total of 30 histological normal or fibrotic mucosa tissues were retrieved from normal subjects or OSF patients recruited from the Department of Dentistry, Chung Shan Medical University Hospital. Normal BMFs and fibrotic BMFs (fBMFs) were cultivated, as previously described [24]. Cell cultures between the third and eighth passages were used.

### 4.3. RNA Sequencing

In order to identify genes that showed differential expression between normal buccal mucosa (N) and OSF tissues, high-throughput RNA sequencing was used to screen for the putative targets. We isolated the total RNA from the clinical samples using a Trizol reagent and the quality of the RNA was assured by the manufacturer of Genomics Inc. After constructing an RNA-seq library, the discrepancies in the transcript levels among the samples were calculated and detected by the FPKM (fragments per kb of transcript per million mapped reads) method, using HiSeq2500 (Illumina, San Diego, CA, USA) [45].

### 4.4. Quantitative Real-Time PCR

The total RNA was extracted with a Trizol reagent and the Superscript III first-strand synthesis system (Invitrogen Life Technologies, Carlsbad, CA, USA) was employed to reverse-transcribe the mRNAs according to the manufacturer’s instructions. qRT-PCR reactions on resulting the cDNAs were performed on an ABI StepOne™ Real-Time PCR System (Applied Biosystems, Foster City, CA, USA).

### 4.5. Western Blot Analysis

Western blot analysis was carried out as previously described [24]. The primary antibodies against Snail, α-SMA, COL1A1, IL-6 and vimentin were purchased from Santa Cruz Biotechnology, Inc. (Santa Cruz, CA, USA). GADPH was used as an internal control.

### 4.6. Immunofluorescent Staining

The cell culture medium was removed and the cells were rinsed with PBS prior to fixation. Cells were then fixed in 4% paraformaldehyde for 10 min. The primary antibody against Snail (Santa Cruz Biotechnology, Santa Cruz, CA, USA) was incubated for 3 h, followed by the corresponding secondary antibody. DAPI was used to indicate the nuclei.

### 4.7. Inhibition and Overexpression of Snail

A lentivirus-delivered short hairpin RNA (shRNA) targeting Snail was performed as follows. The pLV-RNAi vector was obtained from Biosettia Inc. (Biosettia, San Diego, CA, USA). The method of cloning the double-stranded shRNA sequence was described in the manufacturer’s protocol. The oligonucleotide sequence of the lentiviral vectors expressing shRNA that targets Snail was synthesized and cloned into pLV-RNAi in order to generate a lentiviral expression vector.

As for the overexpression of Snail, the cDNA were cloned in pLV-EF1a-MCS-IRES-Puro (BioSettia, Cat. No: cDNA-pLV01; San Diego, CA, USA). Lentivirus production was carried out by the co-transfection of the plasmid DNA mixture with the lentivector plus helper plasmids (VSVG and Gag-Pol) into 293T cells (American Type Culture Collection, Manassas, VA, USA), using Lipofectamine 2000 (LF2000, Invitrogen, Carlsbad, CA, USA).

### 4.8. Collagen Gel Contraction

A mixture of cell and collagen was added into a 24-well plate and incubated until the polymerization of the collagen gel was achieved. Subsequently, the gels were detached from the well using a 200 μL pipet tip and incubated in a 0.5 mL MEMα medium for 48 h. The contraction of the gels was photographed and the contraction index was calculated using ImageJ software (National Institutes of Health, Bethesda MD, USA).

### 4.9. Cell Migration and Invasion Assays

To examine the migration and invasion capacities of these cells, a Transwell system with a polycarbonate filter membrane of 8-μm pore size (Corning, UK) was used. For the invasion assay, Matrigel (BD Pharmingen, NJ, USA) was coated to the membrane. Cells were seeded in the upper chamber with a serum-free medium, followed by 48 h of incubation. Serum-containing media were used as the chemoattractant in the lower chamber. Cells on the other side of the membrane were stained with crystal violet (Sigma-Aldrich) subsequent to fixation. These cells were counted from five different visual areas of 100-fold magnification under a microscope.

### 4.10. Luciferase-Reporter Assay

Luciferase-reporter plasmid was co-transfected with Snail into the constructs. A β-galactosidase-expressing vector was included as an internal control for transfection efficiency. After 24 h, the cells were lysed and both the luciferase and β-galactosidase activities were determined with enzyme assay kits (Promega, Madison, WA, USA). Luminescence was quantitated using a TD-20e luminometer (Turner Designs Inc., San Jose, CA, USA) from duplicate plates. The representative results from three independent experiments were used.

### 4.11. Statistical Analysis

Three replicates of each experiment were performed. Data were expressed as the mean ± SD and analyzed by Student’s *t*-test. *p* < 0.05 was considered statistically significant.

## 5. Conclusions

In summary, we found that Snail was overexpressed in the OSF specimens and fBMFs, which may have been due to the stimulation of the areca nut. Besides this, our data showed that Snail was critical to the maintenance of myofibroblast activities and the upregulation of Snail mediated the differentiation of myofibroblasts, at least in part, via the elevation of IL-6. Most importantly, we revealed that there is a positive feedback loop between IL-6 and Snail during oral fibrogenesis. These findings suggest that therapeutic approaches that target Snail may be able to relieve the pernicious progression of OSF and downregulate the chronic inflammation caused by it. Overall, the results of the present study provide a better understanding of the relationship between areca nut-induced inflammation and EMT progression.

## Supplementary Materials

The following are available online at https://www.mdpi.com/2072-6694/12/6/1611/s1, Figure S1: Original immunoblotting data for Figure 1E, Figure S2: Original immunoblotting data for Figure 2B, Figure S3: Original immunoblotting data for Figure 3A, Figure S4: Original immunoblotting data for Figure 3A, Figure S5: Original immunoblotting data for Figure 4A, Figure S6: Original immunoblotting data for Figure 4A, Figure S7: Original immunoblotting data for Figure 5B, Figure S8: Original immunoblotting data for Figure 5B, Figure S9: Original immunoblotting data for Figure 5B, Figure S10: Original immunoblotting data for Figure 5B, Figure S11: Original immunoblotting data for Figure 5B, Figure S12: Original immunoblotting data for Figure 6A, Figure S13: Original immunoblotting data for Figure 6A, Figure S14: Original immunoblotting data for Figure 6B.
