# Supplementary material for: Positive Feedback Loop of SNAIL-IL-6 Mediates Myofibroblastic Differentiation Activity in Precancerous Oral Submucous Fibrosis
Source: Cancers (Basel). 2020 Jun 18;12(6):1611. doi: 10.3390/cancers12061611 (PMC7352888; doi:10.3390/cancers12061611)
Supplement: Supplementary file 1 [file cancers-12-01611-s001.pdf]

Supplementary Materials

Positive Feedback Loop of SNAIL-IL-6 Mediates Myofibroblastic Differentiation Activity in Precancerous Oral Submucous Fibrosis

Chih-Yu Peng, Yi-Wen Liao, Ming-Yi Lu, Chieh-Mei Yang, Pei-Ling Hsieh and Cheng-Chia Yu

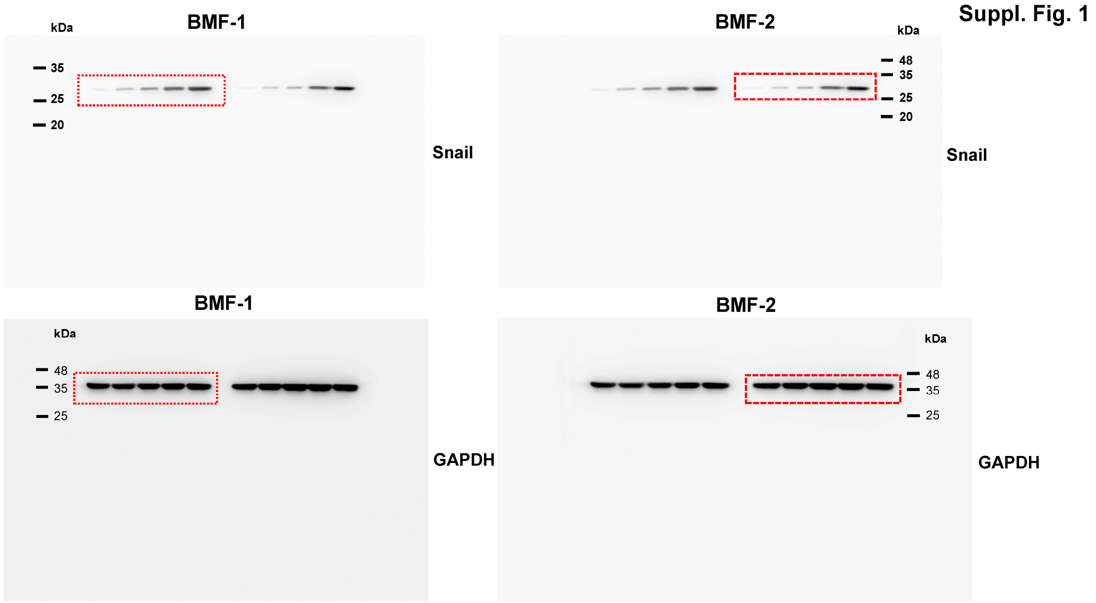

Figure S1. Original immunoblotting data for Figure 1E.

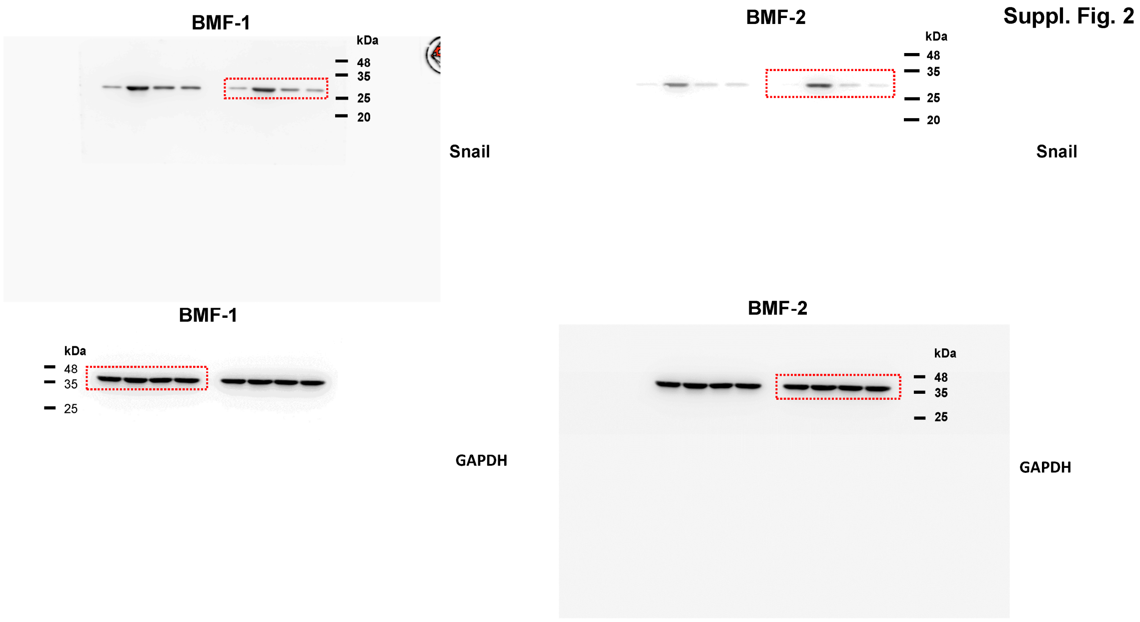

Figure S2. Original immunoblotting data for Figure 2B.

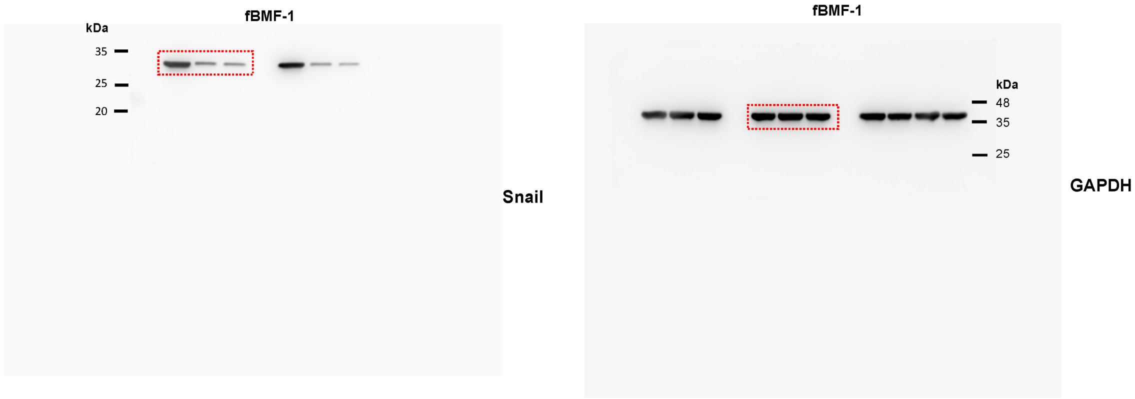

Figure S3. Original immunoblotting data for Figure 3A.

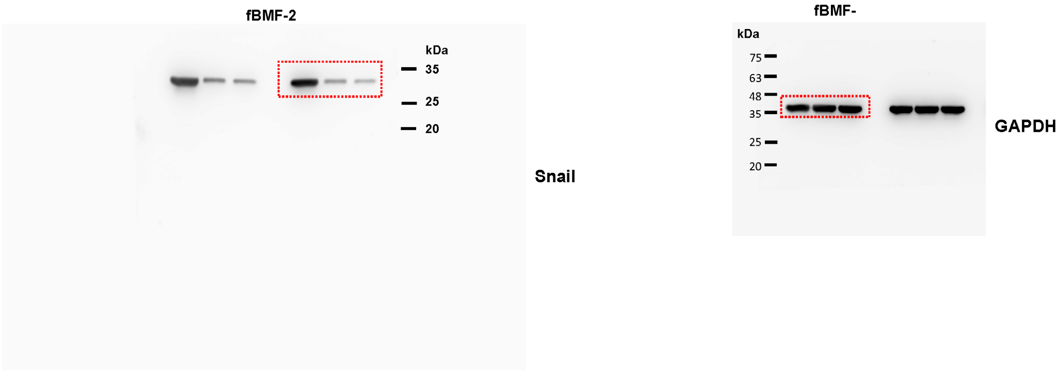

Figure S4. Original immunoblotting data for Figure 3A.

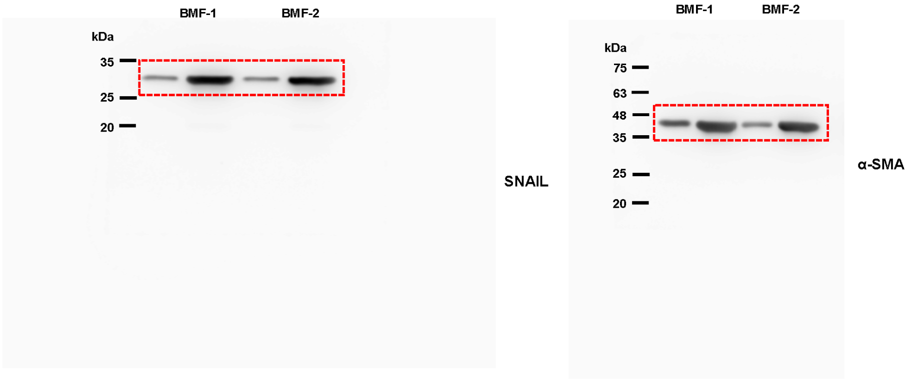

Figure S5. Original immunoblotting data for Figure 4A.

Suppl. Fig. 6

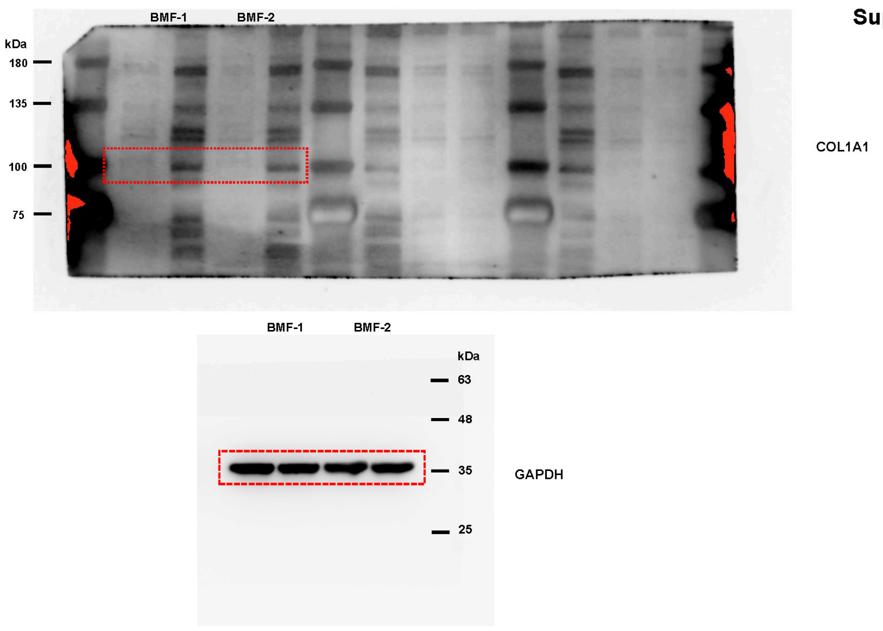

Figure S6. Original immunoblotting data for Figure 4A.

Suppl. Fig. 7

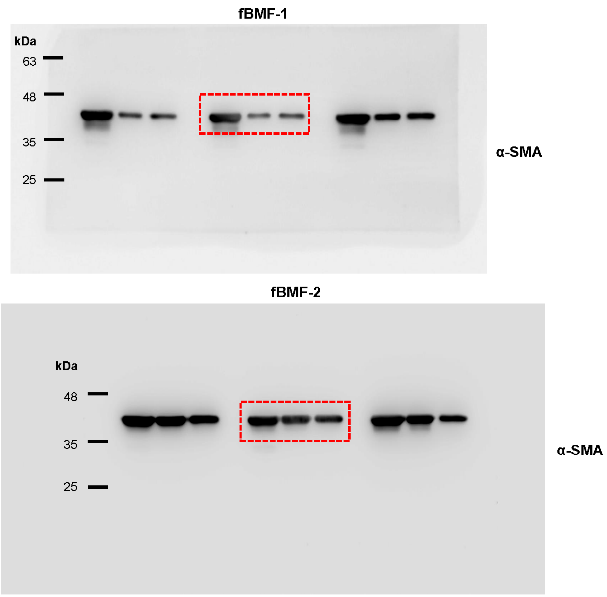

Figure S7. Original immunoblotting data for Figure 5B.

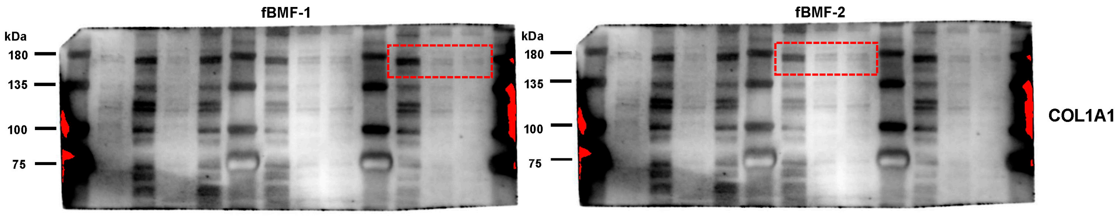

Figure S8. Original immunoblotting data for Figure 5B.

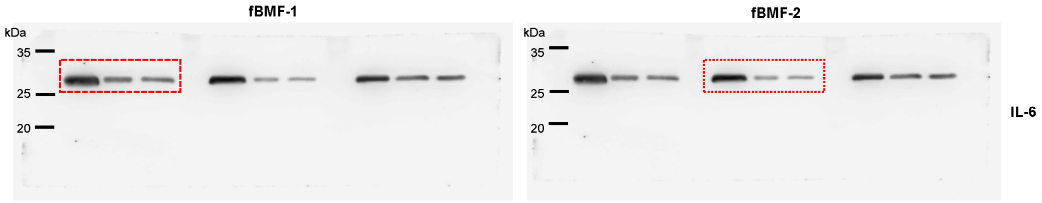

Figure S9. Original immunoblotting data for Figure 5B.

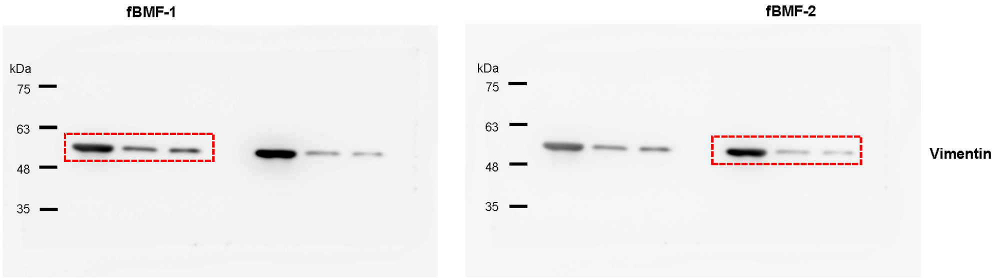

Figure S10. Original immunoblotting data for Figure 5B.

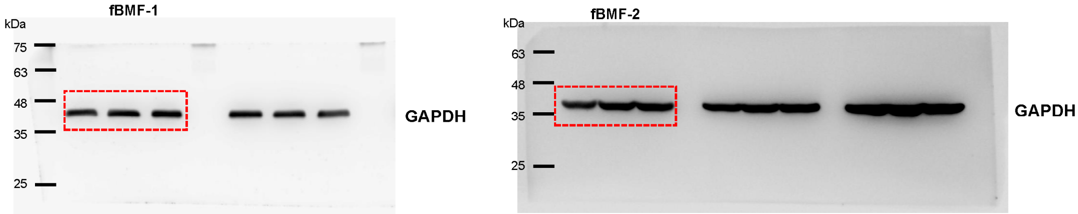

Figure S11. Original immunoblotting data for Figure 5B.

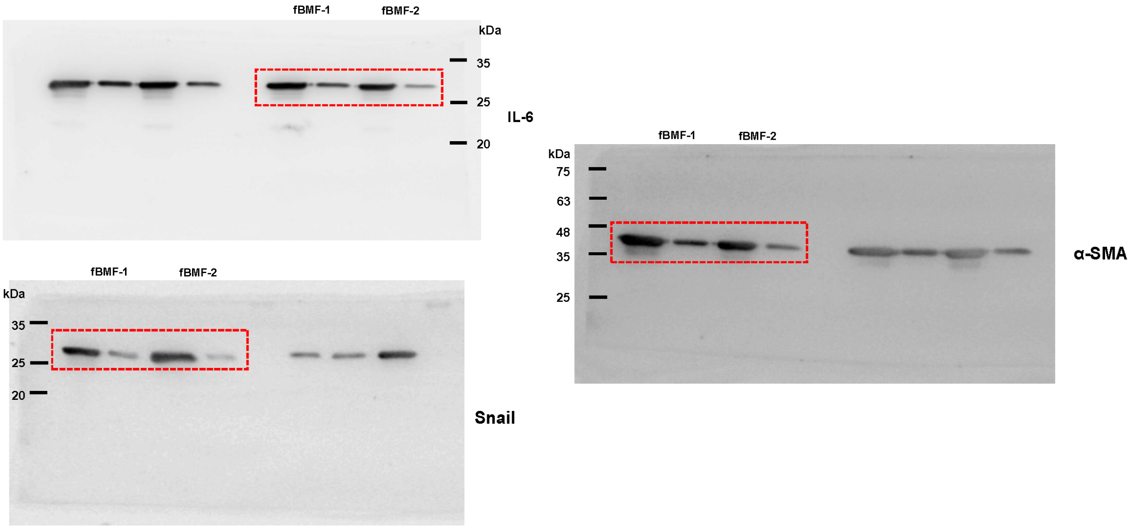

Figure S12. Original immunoblotting data for Figure 6A.

Suppl. Fig. 13

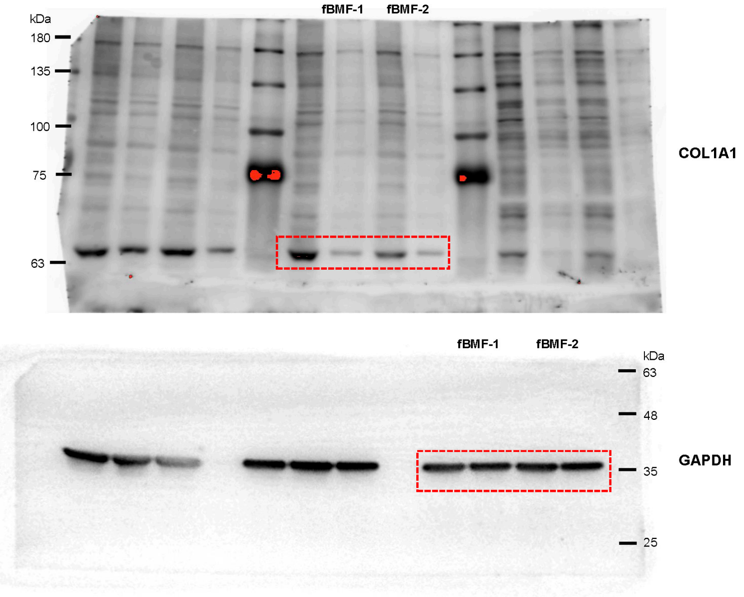

Figure S13. Original immunoblotting data for Figure 6A.

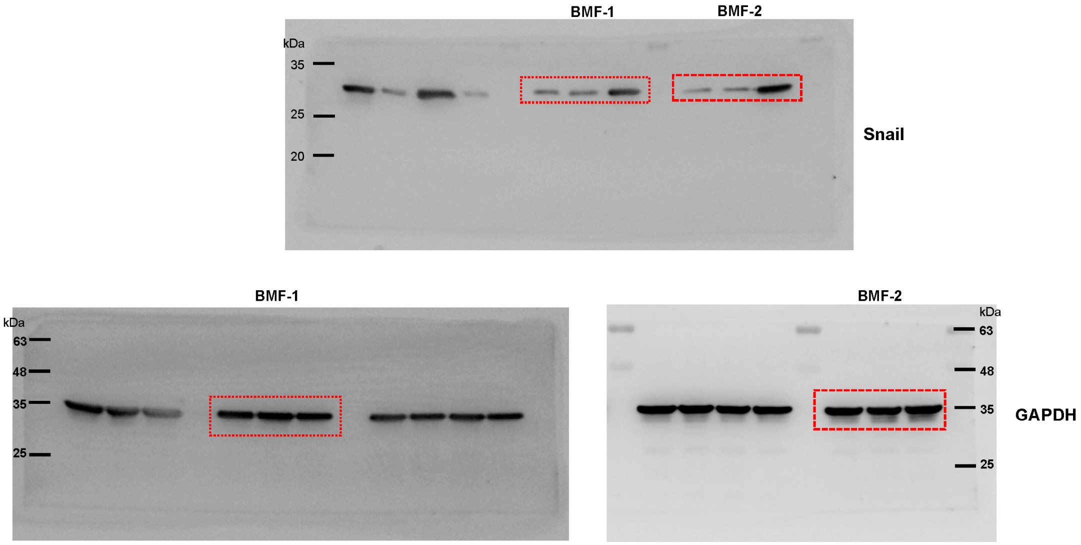

Figure S14. Original immunoblotting data for Figure 6B.

© 2020 by the authors. Licensee MDPI, Basel, Switzerland. This article is an open access article distributed under the terms and conditions of the Creative Commons Attribution (CC BY) license (http://creativecommons.org/licenses/by/4.0/).
